# Quantitative Analysis of the Calcium Hydroxide Content of EVA-Modified Cement Paste Based on TG-DSC in a Dual Atmosphere

**DOI:** 10.3390/ma15072660

**Published:** 2022-04-04

**Authors:** Zhenlei Zhang, Jiang Du, Meilun Shi

**Affiliations:** 1 Key Laboratory of Advanced Civil Engineering Materials, Ministry of Education, Tongji University, Shanghai 201804, China; zhangzhenlei@tongji.edu.cn; 2 School of Materials Science and Engineering, Tongji University, Shanghai 201804, China; 13671751004@163.com

**Keywords:** dual atmosphere, TG-DSC, calcium hydroxide content, hydration degree, EVA

## Abstract

The calcium hydroxide (Ca(OH)_2_) content is one of the main indices of cement hydration degree. In order to accurately determine the calcium hydroxide content of ethylene and vinyl acetate (EVA) copolymer-modified cement paste, a dual atmosphere thermogravimetric method (first in an oxidizing atmosphere and then in an inert atmosphere) was used to track the mass loss and change in enthalpy by TG-DSC (simultaneous thermogravimetry and differential scanning calorimetry). The results showed that using the dual atmosphere thermogravimetric method, the source of mass loss can be distinguished. The exothermic peaks in an oxidizing atmosphere show the oxidation reactions of EVA, while the endothermic peak in an inert atmosphere is due to the pyrolysis reaction of EVA and the decomposition of the calcium hydroxide. The influence of EVA on cement hydration was investigated. The results showed that the polymer powder can be dispersed in water, forming a kind of composite membrane. The test method of dual atmosphere thermogravimetry to measure the calcium hydroxide content of polymer-modified cement pastes is more accurate and convenient than those previously applied.

## 1. Introduction

Hardened cement paste is a composite material whose properties ultimately depend on the components and their spatial relationship to each other. Calcium silicate hydrate (C-S-H) gel, Ca(OH)_2_, ettringite (AFt), and monosulfoaluminate (AFm) are important hydrated products and components of hardened cement pastes [1,2,3,4,5]. They play a crucial role in the various behaviors of hardened cement paste, such as strength and durability. In order to acquire the desired properties, it is important to characterize and quantify these hydrated products of the hardened cement paste, especially for those with various chemical and mineral admixtures. Calcium hydroxide is the second major component of hydrated Portland cement, and it was shown that the percentage of Ca(OH)_2_ is linearly related to the hydration of cement [6,7,8]. Therefore, the hydration degree and the impact of the admixture could be described based on the quantity of formed Ca(OH)_2_. Many studies have been performed on the quantitative analysis of calcium hydroxide (CH) content of cementitious materials [7,9,10,11,12,13]. common methods include chemical extraction, simultaneous thermogravimetry and differential scanning calorimetry (TG-DSC), X-ray diffraction (XRD) methods, etc. The chemical extraction method [14,15] is frequently used for its simplicity and reliability. In recent years, thermal analysis and XRD have been widely used in the content analysis of CH. Ramachandran VS [16] tested the content of CH by separately using chemical extraction and thermal analysis. In his work, a remarkable difference in the content of CH with an increase in cement age was found due to erosion of the extraction agent to C-S-H gel. Although XRD is a commonly used quantitative method, its main disadvantage is that it is only sensitive to crystals, ignoring the existence of some amorphous CH powder in the cement hydration products. In addition, as a hexagonal tabular crystal, the test results interfered with the preferred orientation of CH [17,18]. Zhang TL [19] proposed that the content of CH can be analyzed by thermogravimetry combined with differential scanning calorimetry. The method is rapid and convenient, and the test results were stable and accurate as the environmental and artificial factors were negligible. Although TG-DSC is an effective method in the quantitative analysis of the content of CH, some deficiencies exist, for example, in the hydration products of polymer-modified cement, the negligence of overlapping of the polymer decomposition weight loss peak and calcium hydroxide dehydration peak would introduce experimental error.

For this reason, in order to determine the CH content accurately, we proposed a dual atmosphere thermogravimetric test method to measure polymer-modified cement paste. Ethylene and vinyl acetate copolymer (EVA) as a typical polymeric additive of cement-based materials, was used in this study for its good film forming characteristic [20], corrosion resistance [21], heat preservation, and flexibility. Consideration was given to the physical and chemical effects of polymer on cement mortar and its influence on the microstructure of mortar [22]. It was also noted [23] that the addition of EVA delays the early hydration process of cement and reduces shrinkage stress/strain through inhibiting the formation and transformation of C_4_AH_13_, as well as the formation of AFt and Ca(OH)_2_.

In this paper, to explore the chemical interaction between EVA and Portland cement hydration, we carried out a quantitative analysis of CH in various EVA contents. The dehydration of CH mainly appear in the temperature range from 325 to 490 °C and the curve almost coincided with the degradative peak of EVA in a N_2_ atmosphere. In order to determine the calcium hydroxide content of EVA-modified cement paste accurately, a dual atmosphere thermogravimetric method (first in an oxidizing atmosphere and then in an inert atmosphere) was used to track the weight loss and the change in enthalpy of TG-DSC. 

## 2. Materials and Methods

### 2.1. Materials

In this study, P•I 42.5 Portland cement with a specific surface area of 347 m^2^/kg was provided by China Building Materials Academy, and EVA of 5044N was provided by Wacker Chemical China Co., Ltd; their chemical and mineral compositions and properties provided by the manufacturers are listed in Table 1, Table 2 and Table 3.

### 2.2. Sample Preparation

Five types of specimens were used, with a water to cement mass ratio (w/c) of 0.3, and polymer/cement (m_p_/m_c_) ratios of 0, 0.04, 0.08, 0.12, and 0.16. Samples were casted and cured in a standard curing room (20 °C, 90% RH) for 24 h. Then, the specimens were demolded and allowed to cure in a controlled temperature (20 ± 2 °C) and humidity (65 ± 5%). All tests were performed on the 28th day.

### 2.3. Methods

The simultaneous thermal analysis (TG-DSC) was performed with a type of STA 449C (NETZSCH, Selb, Germany) to measure the mass changes and caloric data. It was operated at a constant ramping rate of 10 ℃/min. A dual atmosphere was proposed, first in an oxidizing atmosphere until 420 °C was reached and then in pure nitrogen to 1000 °C, with the purge gas at a flow rate of 50 mL/min. Crucibles were made of aluminum oxide.

The Fourier transform infrared spectrum (FT-IR) characterization was performed at room temperature with an Equinox 55 spectrometer (Rosenhiem, Germany).

The X-ray diffraction (XRD) measurements were carried out on a diffractometer using Cu Kα radiation at a generation rate of 40 KV and a current of 250 mA (Rigaku, Japan).

The scanning electron microscopy (SEM) was performed on a Quanta 200 (FEI, Fremont, CA, USA) at an accelerating voltage of 15 kV. The samples were sputtered with thin layers of Au.

## 3. Results and Discussion

### 3.1. XRD Pattern

**Figure 1:**
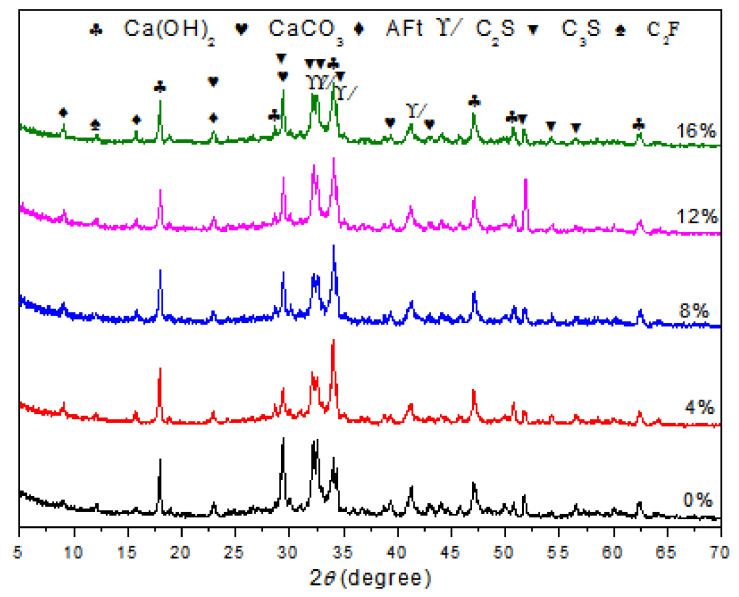
XRD patterns of cement paste and EVA-Modified cement pastes at 28 days of age.

To observe the formation of the hydrates, XRD patterns were recorded at the 28th day for each different polymer/cement ratio of EVA-modified cement paste. The results are shown in Figure 1. It was observed that the characteristic peaks of CH were at 18.08, 34.08, and 47.12°; those of CaCO_3_ were at 29.40, 39.40, and 43.14°; and those of AFt were at 9.09, 15.78, and 22.94°. These results showed the typical hydrated products in hardened cement paste and were consistent with the results from other researches [24,25,26,27]. Although the peak intensity of CH was not directly proportional to the content of CH in hardened cement paste, the content of CH generation could be viewed as an indicative description for the variations in phases upon EVA [28,29]. It was noticed that the peak intensities of CH at around 18.04° presented a slight decrease as the content of EVA increased. The peak intensity was usually related to the content of the corresponding mineral phase. This result showed that the addition of EVA polymer caused a minor delay to the hydration process of cement and reduced the formation of CH.

### 3.2. FT-IR Spectrum

**Figure 2:**
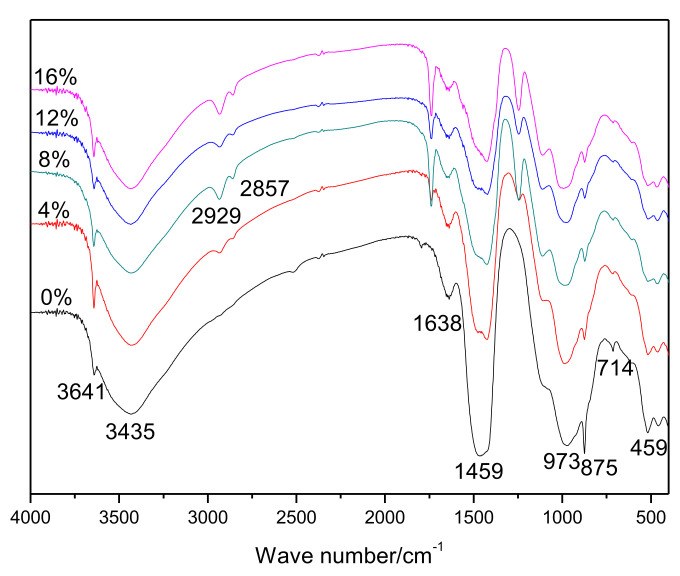
FT-IR spectra of 28-day cured cement pastes.

The results of the FT-IR spectrophotometry of hardened pastes are shown in Figure 2. An interesting result was observed in that the bands at 2929 cm^−1^ and 2857 cm^−1^_,_ which are associated with the EVA [25], gradually became stronger as more polymers were added. The bands at 3641, 1459, and 714 cm^−1^ were related to the presence of CH [26]. The band at about 1638 cm^−1^ was related to the formation of Aft [30], and the bands at 973 cm^−1^ and 875 cm^−1^ were attributed to hydrated calcium silicates [31] and carbonate [31], respectively.

### 3.3. SEM Observation

**Figure 3:**
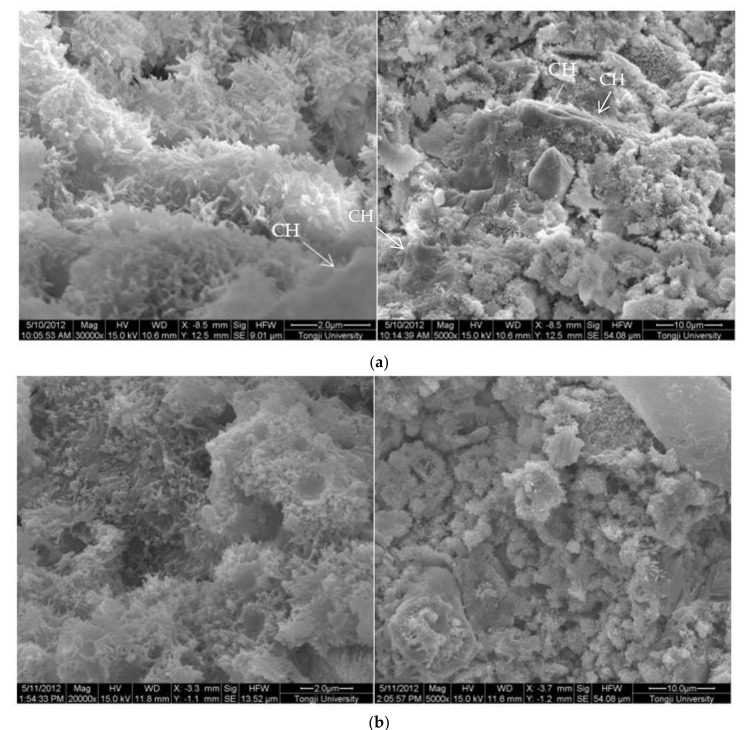

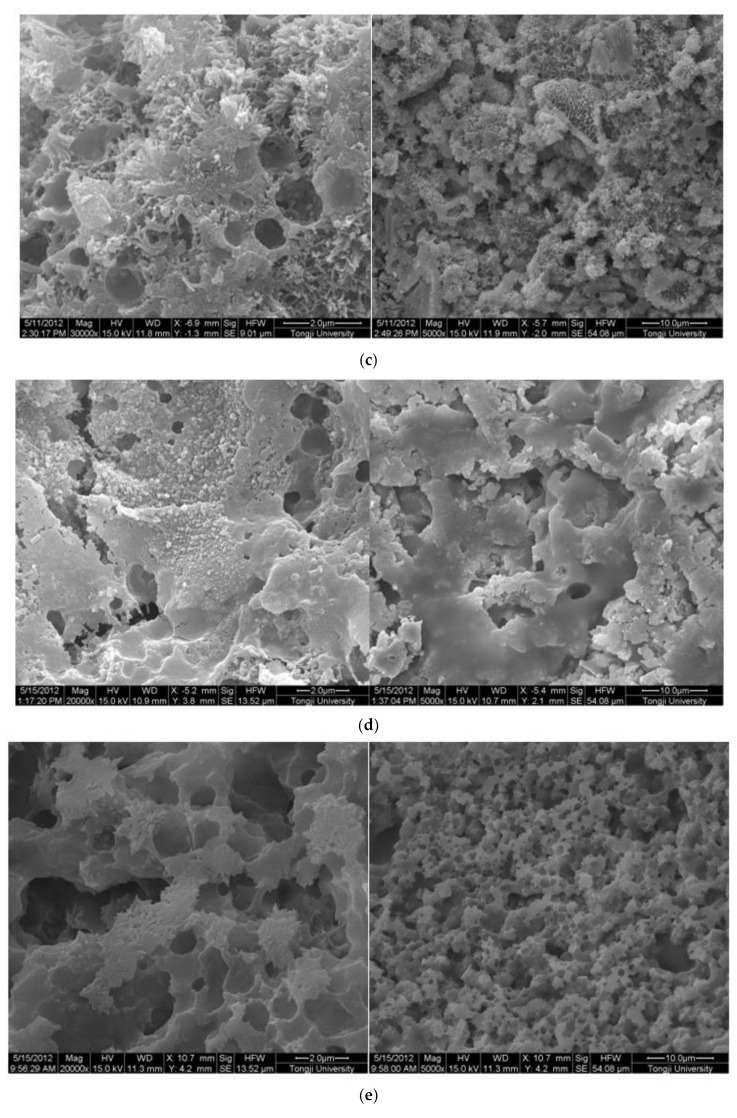
Morphology of 0.3 w/c cement paste and EVA-modified cement pastes with different EVA latex/cement ratios at 28 days. (**a**) 0.3 w/c cement paste, (**b**) m_p_/m_c_ = 4%, (**c**) m_p_/m_c_ = 8%, (**d**) m_p_/m_c_ = 12%, (**e**) m_p_/m_c_ = 16%.

The SEM images of 0.3 w/c hardened paste and pastes with different contents of EVA are shown in Figure 3. It can be seen from Figure 3 that AFt and calcium hydroxide with various morphologies were found in the hardened pastes, and they were attached to the surface of the films or filled in the pores (Figure 3b), but not in the EVA-Modified mortars with a m_p_/m_c_ higher than 8% (Figure 3c–e). Obviously, the polymer films were relatively weak at the m_p_/m_c_ of 4%, and narrow gaps were found and the hardened paste presented with a loosened microstructure. However, a different case was also observed in that the polymer films became thicker as more polymers were mixed (as shown in Figure 3c–e). Usually, the polymer films were related to the ratio of polymer to cement. More polymers implied thicker films. In addition, when the m_p_/m_c_ was 8%, perfect mesh network polymer films were observed, and more small pores were observed as more EVA was added. With the m_p_/m_c_ growing, the thickness and proportion of the mesh network films increased, and the organic–inorganic interpenetrating structures were formed.

### 3.4. Calcium Hydroxide Content from TG-DSC

**Figure 4:**
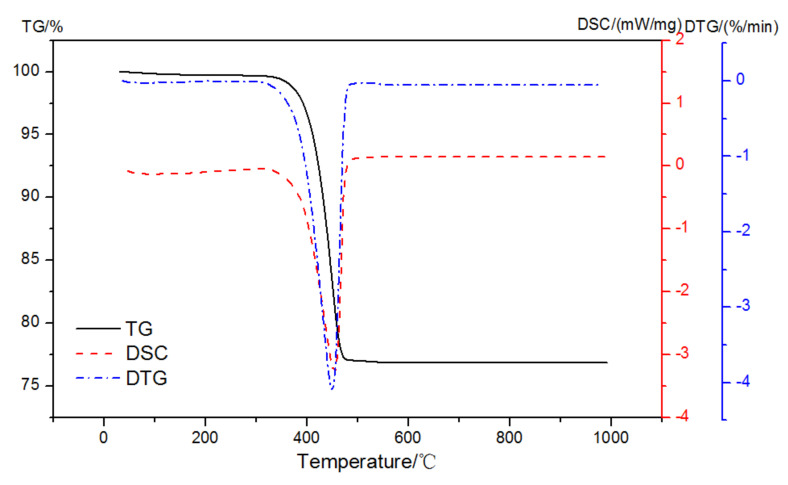
TG-DSC-DTG curves of analytically pure CH in a pure N_2_ gas dynamic atmosphere.

The mass loss and DSC curves of analytically pure Ca(OH)_2_ in pure N_2_ gas dynamic atmosphere are shown in Figure 4. There was only one prominent peak observed at 350~500 °C that was related to the dehydroxylation of Ca-OH according to Equation (1):

(1) $$C_{a}{\left(OH\right)}_{2}\to C_{a}O+H_{2}O$$

**Figure 5:**
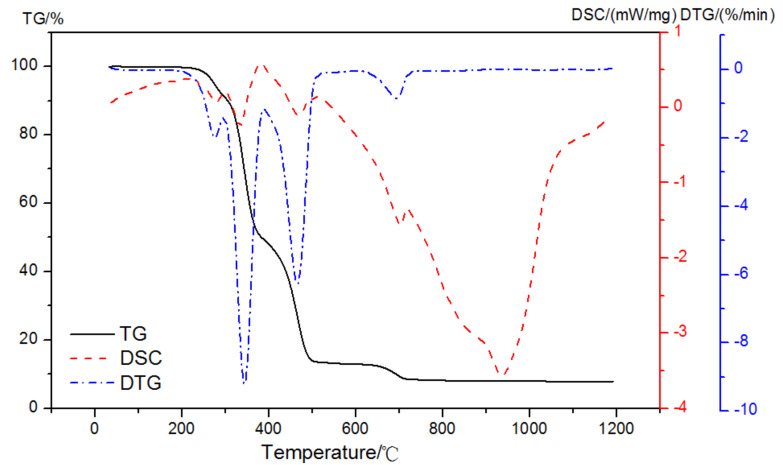
TG-DSC-DTG curves of EVA in a pure N_2_ gas dynamic atmosphere.

As shown in Figure 5, there were four prominent peaks observed in the DTG curve of EVA5044 in a pure N_2_ gas dynamic atmosphere. The two primary mass loss peaks were at 295~383 °C and 383~510 °C. The other peaks were at 208~295 °C and 630~720 °C. DSC curves corresponding to each weight loss process were endothermic peaks. It was obvious that the dehydration of CH and the decomposition of EVA both appeared at the temperature region of 350 °C to 500 °C in the atmosphere of nitrogen. When analyzing the content of CH according to Equation (1), the loss mass was difficult to calculate because of the overlapping of thermal decomposition. 

**Figure 6:**
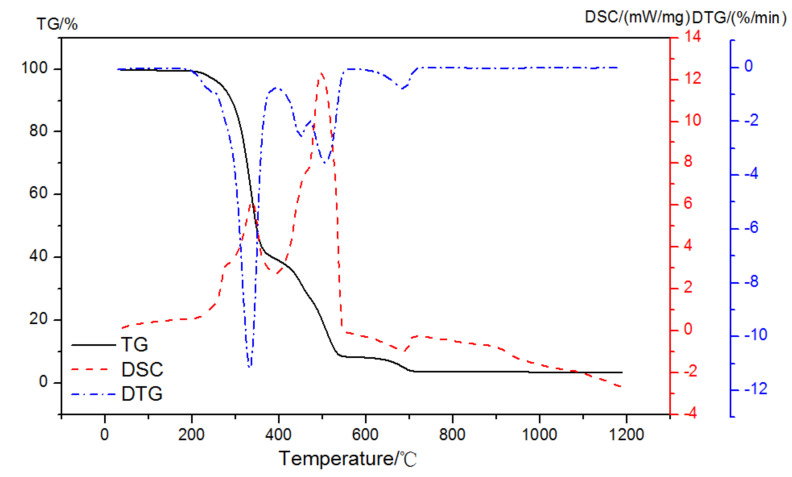
TG-DSC-DTG curves of EVA in an air atmosphere.

When the atmosphere was switched to air, three exothermic peaks and one endothermic peak might be observed with mass loss peaks at 210~395 °C, 395~467 °C, 467~550 °C, and 610~710 °C, as illustrated in Figure 6. Therefore, the enthalpy change in the first exothermic peak in the oxidation reaction could be used to analyze the amount of EVA, and then its weight loss in the corresponding stage could be calculated.

**Figure 7:**
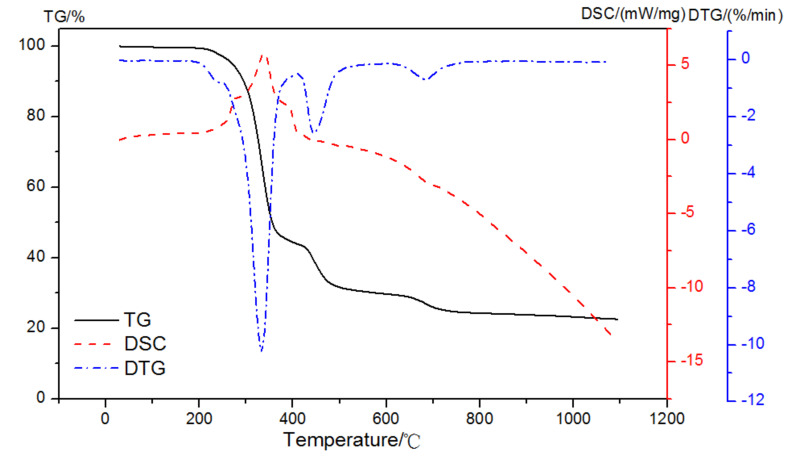
TG-DSC-DTG curves of EVA in a dual atmosphere.

To prevent an endothermic and an exothermic process appeared at the same time, a dual atmosphere was used. With an oxidizing atmosphere up until 420 °C and then a pure nitrogen to 1000 °C with the purge gas at a flow rate of 50 mL/min, the TG-DSC-DTG result of EVA in dual atmospheres is shown in Figure 7. There were three distinct regions in the heating of the pastes: up to 420 °C in the process of an exothermic reaction with the reaction enthalpy of 697 J/g; then, the pyrolysis reaction of EVA took place in the nitrogen atmosphere, and the mass loss was 12.06% between 420 and 510 °C; and, finally, it turned to the range of 620~740 °C with 4.70% mass loss. The content of EVA and the corresponding weight loss could be calculated according to the exothermic peak of the oxidation reaction in the air atmosphere up to 420 °C.

**Figure 8:**
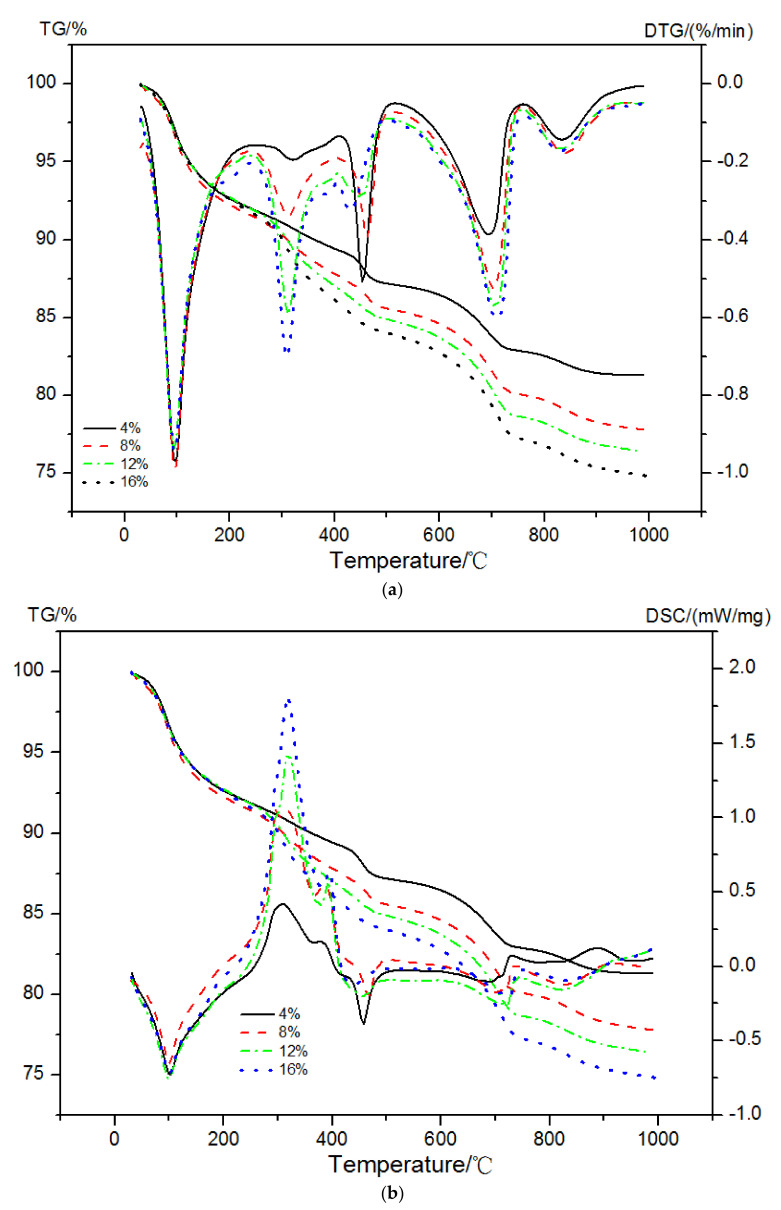
TG-DTG and TG-DSC curves of EVA-modified cement pastes. (**a**) TG-DTG, (**b**) TG-DSC.

The mass loss, DTG curves, and enthalpy changes in EVA-Modified cement pastes in a dual atmosphere TG-DSC method are shown in Figure 8. The peak at a temperature of 30 °C to 250 °C was due to the decomposition of AFt/AFm and C-S-H gels. The second peak occurring at a temperature between 250 °C and 390 °C mainly originated from the oxidation reaction of EVA and decomposition of C-S-H gels. The dehydration of CH and the decomposition of EVA together led to the third peak between 450 °C and 550 °C in the DTG curves. The last peak at a temperature range of 650~800 °C was caused by the decarbonation behavior of calcium carbonates (CaCO_3_).

**Figure 9:**
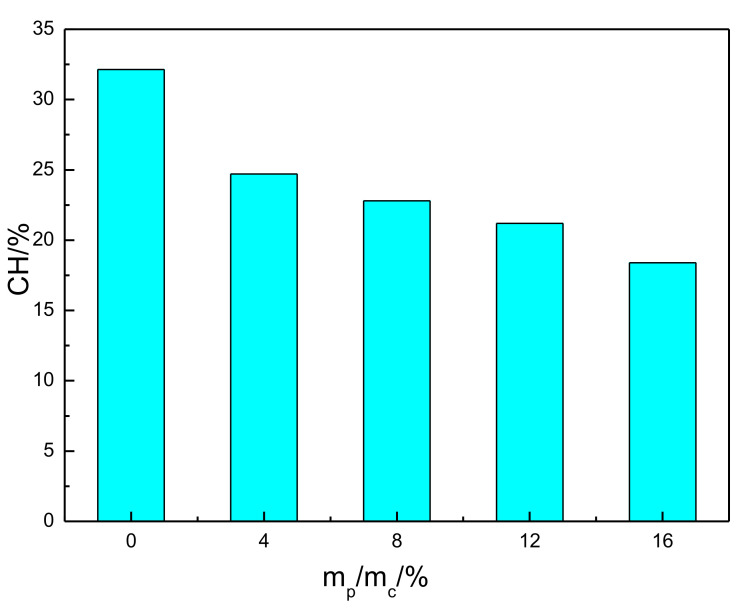
Calcium hydroxide content of EVA-Modified cement pastes.

The calcium hydroxide content was determined using Equation (2):

(2) $$M_{CH}=\frac{74.09\left(M_{loss}-M_{EVA}\right)}{18.02M_{sample}}$$

where *M_CH_* was the mass of CH (wt%), *M_sample_* was the weight of the sample, *M_loss_* was the mass loss at 420~510 °C, *M_EVA_* was the mass loss of EVA at 420~510 °C, 74.09 is the molar weight of calcium hydroxide, and 18.02 is the molar weight of water. The content of CH in the cement pastes was calculated, and the corresponding results are presented in Figure 9. It can be seen that the CH content in hardened paste decreased with the addition of EVA. As observed in Figure 3, EVA adsorption on the surface of hydrated products was inhibited even further in the hydration process and slowed down the hydration rate and the overall reaction.

## 4. Conclusions

TG-DSC was a very suitable technique to analyze the CH content of cement paste. The dual atmosphere method was established in order to distinguish the source of the mass loss. The experiments showed that EVA had a significant influence on the structure and hydration degree of cement pastes. Larger amounts of EVA could cause an even slower hydration rate.

## Figures and Tables

**Table 1:** The chemical compositions of Portland cement used in this study (wt, %).

Oxides	SiO_2_	CaO	Al_2_O_3_	Fe_2_O_3_	MgO	SO_3_	f-CaO	Na_2_O	Cl^−^	Loss
Content/%	22.01	62.1	4.00	3.47	2.57	2.71	0.87	0.53	0.01	1.73

**Table 2:** The mineral compositions of Portland cement used in this study (wt, %).

Mineral Compositions	C_3_S	C_2_S	C_3_A	C_4_AF
Content/%	57.34	18.90	6.47	11.25

**Table 3:** The detailed properties of EVA used in this study.

Solid	Ash Content/%	Glass Transition Temp./°C	Min. Film Building Temp./°C	Granule Size/μm
98~100	8–12	−6	0	1–7

## Data Availability

Data sharing is not applicable for this paper.
